# ADHD and Sex Hormones in Females: A Systematic Review

**DOI:** 10.1177/10870547251332319

**Published:** 2025-04-18

**Authors:** Elyssa Osianlis, Elizabeth H. X. Thomas, Lisanne Michelle Jenkins, Caroline Gurvich

**Affiliations:** 1 HER Centre Australia, Department of Psychiatry, School of Translational Medicine, Monash University, Melbourne, VIC, Australia

**Keywords:** ADHD, females, hormones

## Abstract

**Objective:**

ADHD is a neurodevelopmental condition associated with elevated symptoms of inattention and hyperactivity/impulsivity. Symptoms of ADHD typically persist into adulthood and can impair functioning and overall quality of life. In females (including women and people assigned female at birth), ADHD is under-recognized, and knowledge about the relationship between ADHD symptoms and sex hormones is lacking. This systematic review aimed to synthesize the current evidence investigating the relationship between ADHD symptoms (including medication effects) and sex hormones in females.

**Method:**

Searches were conducted within Ovid MEDLINE, Embase, and PsycINFO from 1980 to January 2025. Included studies investigated ADHD symptoms in the context of hormonal changes in females, including studies specifically exploring ADHD and sex hormones, as well as hormonal life stages (puberty, menstrual cycle, and pregnancy). Narrative synthesis was utilized for data extraction, grouping studies by hormonal phase.

**Results:**

A total of 11 studies were included in this review. Evidence was largely suggestive of a relationship between ADHD symptoms and sex hormones in females, specifically in puberty and across the menstrual cycle. Findings were limited by the small number of studies reviewed, often with small sample sizes and considerable diversity in participant populations and outcome measures.

**Conclusion:**

Sex hormones and phases related to hormonal changes (such as puberty and the menstrual cycle) may be associated with ADHD symptom changes in females. Further research is needed to understand the relationship between sex hormones and ADHD symptoms and requires investigation of a wider range of hormonal milestones in females, including menopause.

## Introduction

ADHD is a neurodevelopmental condition characterized by symptoms of inattention and hyperactivity/impulsivity (American Psychiatric Association, 2013). ADHD has historically been considered a male condition due to the higher diagnostic rates in boys, with current prevalence estimates in childhood of a 2.4:1 ratio of boys to girls (Polanczyk et al., 2007). More recently, sex and gender^1^ differences in ADHD have been recognized, and demonstrate a likely underdiagnosis of ADHD in females^2^ in childhood and adulthood, rather than a male disposition to ADHD (Faraone et al., 2024; Young et al., 2020). Females typically present with internalizing symptoms of ADHD including inattention, as well as additional symptoms not included in the diagnostic criteria but associated with ADHD, such as executive dysfunction and emotional dysregulation (P. Quinn & Wigal, 2004; Young et al., 2020). Alternatively, males, and particularly younger males/boys present with more externalizing symptoms including hyperactivity; as these symptoms are more observable to teachers and caregivers, they may reinforce sex-based perceptions of ADHD being more common in males, as they conform with typical characterizations of ADHD based on males (Mowlem et al., 2019; Young et al., 2020). In this sense, the traditional conceptualization of ADHD centered around male presentation is challenged by presentation in females, and contributes to the under recognition of ADHD in females. Comorbid mental health symptoms are also highly prevalent in people with ADHD (Choi et al., 2022), and specifically females with ADHD, which may further contribute to misdiagnosis and underdiagnosis of ADHD in females (Ottosen et al., 2019; Young et al., 2020).

Interest has recently grown regarding sex differences in ADHD, including research specifically exploring ADHD presentation and underlying mechanisms in females. Endogenous sex hormones have been identified as one factor that may contribute to the sex differences in ADHD symptoms. Hormones such as estrogen and progesterone are thought to play a key role in cognition and many psychiatric and neurodevelopment conditions (Gurvich et al., 2018). In females, fluctuations of estrogen and progesterone have been directly implicated in conditions including premenstrual dysphoric disorder, postpartum depression, and menopausal depression (Hantsoo & Epperson, 2015; Kulkarni et al., 2024). Other mental health conditions such as schizophrenia have also shown hormonal effects, with exacerbation of symptoms at times of low estrogen, and demonstrated improvement of symptoms with hormonal therapy (Brzezinski et al., 2017).

**Figure 1.**
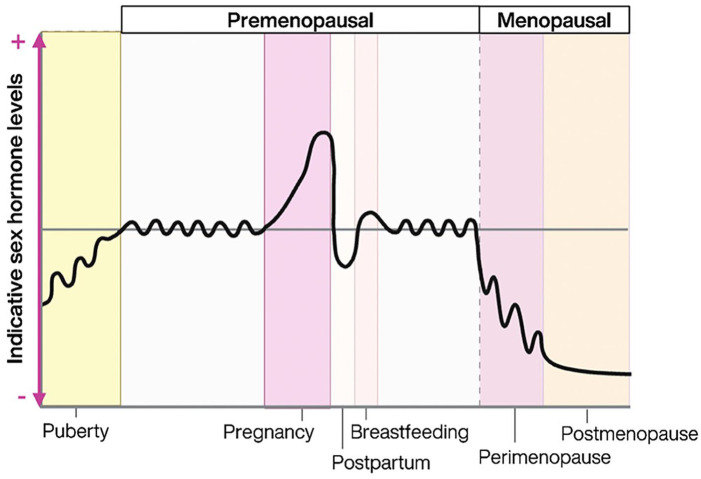
Indicative sex hormone (estrogen and progesterone) levels across the lifespan in females. Created in Biorender.com.

Sex hormones are largely produced by the gonads and primarily associated with their role in reproductive functioning, however they also have important functions as neurosteroids. Estrogen exists in three key forms in females: estrone (E1; primary estrogen during menopause), estradiol (E2; primary estrogen during reproductive years), and estriol (E3; primary estrogen during pregnancy). Progesterone is the second key sex hormone in females and follows the same patterns of estrogen, increasing from childhood into reproductive years, and falling to very low levels in menopause, shown in Figure 1. Androgens such as testosterone are also present in females, though estrogen and progesterone are considered the key hormones in females. Estrogen and progesterone act directly on the hypothalamic-pituitary-adrenal (HPA) axis to modulate release of hormones, and effect regulation of monoamines including serotonin, dopamine, and noradrenaline, which are involved in cognition and behavior (Del Río et al., 2018).

Both preclinical and clinical studies have demonstrated a key role for sex hormones in cognition. Animal studies have shown organizational effects of estradiol to enhance memory, suggesting estradiol activates signaling pathways which stimulate synaptogenesis and changes to neuronal structures that improve functioning (Finney et al., 2020; Luine, 2014). Clinical studies provide evidence to support a role of hormones in cognition; surgical menopause following bilateral oophorectomy with a rapid decrease in endogenous estrogen and progesterone, has been associated with a subjective decline in cognition 6 months later (Chang et al., 2021), as well as an increased risk of later life cognitive decline and dementia (Georgakis et al., 2019; Rocca et al., 2021). Natural menopause, which is associated with a 4- to 10-year period of fluctuating and declining levels of estradiol and progesterone, is often associated with cognitive complaints and symptoms of brain fog, particularly impacting attention and memory (Conde et al., 2021; Epperson et al., 2015; Khadilkar & Patil, 2019).

Dopamine is one of the most recognized neurotransmitters implicated in ADHD pathology and has demonstrated interactions with estrogen and progesterone. In preclinical studies, estrogen has been shown to stimulate dopamine production and reduce reuptake and degradation at the synapse (Del Río et al., 2018). Progesterone however has shown more varied effects; in estrogen environments, progesterone may increase dopamine synthesis in the striatum, however in the prefrontal cortex, allopregnanolone, a progesterone metabolite, may have inhibitory effects on dopamine release (Dazzi et al., 2002; Matuszewich et al., 2000). Theories surrounding a hormonal influence on ADHD suggest sex hormones are likely to modulate neurotransmitters including dopamine, as well as serotonin and noradrenaline (Haimov-Kochman & Berger, 2014). This may provide a mechanism to explain exacerbation of ADHD symptoms during periods of hormonal fluctuation, such as menopause and across the menstrual cycle (Haimov-Kochman & Berger, 2014). However, empirical research investigating the relationship between sex hormones and ADHD symptoms is lacking- and potential confounders such as exogenous hormones (including menopausal therapies and contraceptives) are often not considered in research investigating ADHD in females. Two recent studies in children with ADHD have found associations with gene variances and levels of estrogen receptors (Lin et al., 2023; Sahin et al., 2018). While this is suggestive of a hormonal influence, more evidence is needed. Investigation of ADHD symptoms and medication efficacy in the context of the hormonal milieu experienced by females is necessary to improve understandings of ADHD in females.

The aims of this systematic review are to determine potential effects of female sex hormones on ADHD symptoms/ADHD medication efficacy. Specifically, this review aims to:

(1) Determine potential effects of sex hormones (either measured directly or explored through life phases reflecting hormonal changes, such as puberty, pregnancy, and menopause) on ADHD symptoms or perceived efficacy of ADHD medication in females.
(2) Determine potential effects of female sex hormones (including hormonal life phases) on co-existing mental health symptoms in females with ADHD.

## Methods

This systematic review was completed according to PRISMA 2020 guidelines (Page et al., 2021) and registered on PROSPERO (CRD42024554464). Studies investigating relationships between female sex hormones and ADHD symptoms, published in English in peer-review journals were considered for inclusion. Relevant studies were identified by search of electronic databases Ovid MEDLINE, Embase, and PsycINFO from 1980 to January 24th 2025. Search terms for initial title and abstract screening included terms for ADHD, female sex hormones, hormonal life phases in females (i.e., puberty, pregnancy, and menopause), and synonyms of these terms, as shown in Table 1.

**Table 1.** Search Terms.

Attention Deficit Disorder with Hyperactivity
ADHD
attention deficit hyperactivity disorder
attention deficit disorder
Amphetamines
(clonidine and hormon*)
Atomoxetine Hydrochloride
Methylphenidate
methylphenidate
Lisdexamfetamine Dimesylate
lisdexamfetamine
Dextroamphetamine
dexamfetamine
Guanfacine
guanfacine
Estrogens
estrogen
oestrogen
estrad*
Progesterone
Progest*
testosterone
sex hormone*
Gonadal Steroid Hormones
gonadal steroid hormone*
Menarche
menarche
Menstrual Cycle
Puberty
pubert*
(hormon* and pregnan*)
Contraceptive Agents
contracepti
Hormonal Contraception
Contraceptive Agents, Female
menopause
Menopause
Postpartum Period
postpartum

### Selection Criteria and Search Strategy

Studies were considered to meet inclusion criteria if (i) they included a sample of females/women with ADHD or a proxy ADHD screener, (ii) outcomes included a measure of ADHD symptoms (either a scale score or qualitative report), and (iii) ADHD symptoms were measured in the context of a hormonal phase/environment. Articles were excluded for one or more of the following reasons: (i) there was no all-female group and (ii) it was not a peer reviewed journal article.

All methods of ADHD diagnosis/proxy were included in this review, including formal diagnosis by a clinician, as well as self-reported scale scores indicating ADHD traits. ADHD outcome measures in relation to hormonal phases were included in the summary tables. When available, ADHD medication usage by participants was also recorded.

Studies were first screened by their title and abstract by one author, and then examined at the full text level by two authors. Conflicts between authors were resolved by decision of a third author.

### Data Collection and Analysis

Data extraction was completed by one author for each report. We primarily focused on ADHD measures in relation to a hormonal phase (e.g., menstrual cycle and puberty). Secondary outcome data was collected as mental health symptom measures in relation to the hormonal phase. Results relevant for each outcome domain were included in analysis. Studies were grouped by the hormonal phase within which ADHD symptoms were being measured, including menstrual cycle, puberty, and pregnancy. Studies which could not be grouped due to diversity of hormonal phases were included in a fourth “other hormonal environment” group. Data synthesis was achieved using textual narrative synthesis.

### Study Quality and Risk of Bias

Risk of bias and assessment of study quality was completed for each study included in this review. Risk of bias was determined based on an adapted version of the Newcastle-Ottowa Quality Assessment Scale, with scoring criteria including selection, comparability and outcome of each study (Supplemental Material 1). Classification of bias risk was scored on a scale of 0 to 9: 0 to 4 = unsatisfactory, 5 to 6 = satisfactory, 7 to 8 = good, and 9 = very good. Risk of bias assessment was completed by two authors, with conflicts resolved by decision of a third author.

## Results

### Search Results

**Figure 2.**
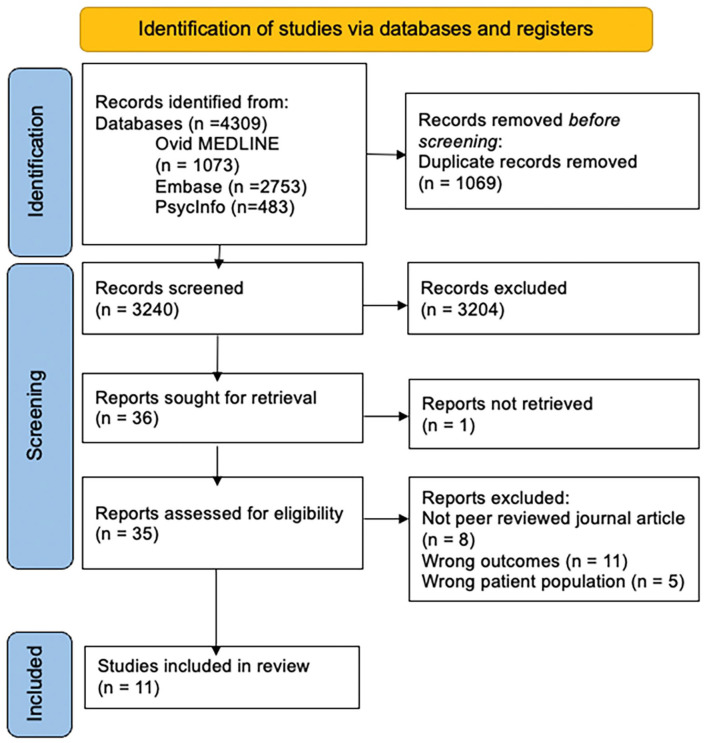
PRISMA flowchart of study inclusion.

The initial search yielded 4,309 records, with duplicate records removed before screening (see Figure 2). Of these, 3,240 records progressed to title and abstract screening, which resulted in exclusion of 3,204 records which did not meet inclusion criteria. Therefore, 35 articles advanced to full text screening, of which 24 were excluded. This resulted in a final *n* = 11 studies included in this review. Studies were grouped according to hormonal life phase investigated, including Menstrual Cycle, Puberty, and Pregnancy, with remaining articles grouped as Other Hormonal Environment.

### Puberty

Of the 11 studies, 3 studies investigated the relationship between puberty and ADHD symptoms in females (summarized in Table 2). Eng et al. (2023), over-sampled a group of 324 girls with ADHD (*M*_age_ = 9.62) and prospectively followed them over 8 years (*N* = 128, *M*_age_ = 15.67). They found that ADHD symptoms of inattention and impulsivity were not associated with pubertal development, whilst hyperactivity significantly decreased in within-person analyses as pubertal development progressed. Depression was significantly increased in females with later pubertal development stages in between-persons analyses, and found to be associated with older age at time of study entry. Ostojic and Miller (2016) found inattention and difficulties in emotional regulation scores were associated with early pubertal onset in college studies. However, this study utilized retrospective ratings of pubertal development relative to peers in a community sample, without any formal assessment of ADHD diagnosis. Conversely, Yuan et al. (2024), reported no significant associations between early menarche and ADHD as assessed by the Adult ADHD Self Report scale in a cohort of school-aged Chinese females.

**Table 2.** Puberty and ADHD.

Author, year, study type	Sample characteristics	Age mean (*SD*)	ADHD medication use	ADHD diagnosis method	ADHD symptom × hormone measure	Results	Risk of bias
Eng et al. (2023) *Prospective cohort study*	324 females year 1 (7–13 years) followed to 128 females year 8 (14–18 years), in a cohort over-sampled for ADHD	Wave 1: 9.62 (1.58), Wave 8: 15.67 (0.98)	Not reported	46% in Wave 1 and 38.28% in Wave 8 ADHD diagnosed (via decision by clinical diagnostic team)	Kiddie Schedule for Affective Disorders and Schizophrenia (KSADS) ADHD Section across pubertal stages (Pubertal Development Scale)	- **Inattention**: no significant effects of pubertal development on inattention symptoms - **Hyperactivity**: hyperactivity symptoms decreased in within-person analyses as females developed pubertally (estimate = −0.19, *SE* = 0.08, *p* ≤ .05) - **Impulsivity**: no significant changes in impulsivity symptoms across puberty - **Mental health symptoms**: Between-persons analyses at study entry showed higher depressive symptoms in females at later pubertal stages, though this was not reflected in within-persons analyses (no difference). Older age at study entry was associated with greater likelihood to experience an increase in depressive symptoms as participants aged.	8, good
Ostojic and Miller (2016) *Cross-sectional study*	253 female, non clinical sample	20.19 (1.69)	Not reported	N/A	Barkley Adult ADHD Rating Scale-IV scores in relation to Pubertal Development Scale-Retrospective Version scores	- **Inattention:** inattention scores were predictive of early pubertal onset (odds ratio = 1.270, 95% CI [1.040, 1.552]) - **Mental health symptoms:** Difficulties in emotional regulation (DERS Impulse Score) were predictive of early pubertal onset	5, satisfactory
Yuan et al. (2024) *Longitudinal, observational study*	1,039 females at Time 1 (baseline), 946 females at Time 2 (1-year follow-up)	12.49 (0.97) at baseline	Not reported	Adult ADHD Self Report Scale (ASRS)	ADHD per ASRS in relation to self-reported age of menarche, and ADHD per ASRS in relation to menstrual regularity (determined by 1–3 Likert scale of self-perceived regularity, length of period, menstruation interval, bleeding duration, menstrual pain)	- **Early menarche and ADHD:** no significant associations between early menarche and incidence rates of ADHD - **Menstrual irregularity and ADHD**: significant association between menstrual irregularity and rates of persistent ADHD (at 1 year follow up; odds ratio = 3.40, 95% CI [1.67, 6.93], *p* < .01)	3, unsatisfactory

### Menstrual Cycle

Of the 11 studies, 4 studies investigated the relationship between the menstrual cycle and ADHD symptoms (summarized in Table 3). Three of the four articles included measured ADHD symptom changes across the menstrual cycle according to participants’ responses to interview questions (Bürger et al., 2024; de Jong et al., 2023; P. O. Quinn, 2005). Of these, one study (P. O. Quinn, 2005) reported a case of a single female participant with a recent ADHD diagnosis, describing decreased perceived efficacy of stimulant medication premenstrually. The remaining two studies used qualitative interviews to derive results from cohorts of participants with ADHD diagnoses. de Jong et al. (2023), recruited nine participants who experienced premenstrual worsening of ADHD symptoms, and tracked within-person effects of increased psychostimulant dosage premenstrually in improving these symptoms. All participants reported improvement of ADHD and mood symptoms (emotional regulation and energy levels), with increased psychostimulant dosage premenstrually after 6 to 24 months (de Jong et al., 2023). Similarly, Bürger et al. (2024), measured perceived association of ADHD symptoms across the menstrual cycle, using reflexive thematic analyses of a cohort of 10 participants diagnosed with ADHD, taking stimulant medication and not on hormonal birth control. Key findings indicated participants perceived changes in ADHD severity and stimulant medication efficacy with their menstrual cycle, specifically effecting symptoms of emotional dysregulation, executive dysfunction, inattention, and concentration difficulties. Symptoms were perceived to change severely, particularly during the mid-luteal phase and menses, with additional negative impacts to quality of life.

**Table 3.** Menstrual Cycle and ADHD.

Author, year, study type	Sample characteristics	ADHD medication use	Age	ADHD diagnosis method	ADHD symptom × hormone measure	Results	Risk of bias
Bürger et al. (2024) *Qualitative interview*	*N* = 10, assigned female at birth	*N* = 10 taking central stimulant medication for ADHD	Median:27, Range 23–39	Official ADHD diagnosis as reported by participants	Interviews with inductive approach regarding ADHD symptoms across menstrual cycle	- **Menstrual phases and ADHD symptoms:** participants perceived associations between menstrual cycle phases and ADHD symptom severity, particularly an increase during midluteal phase and menses - **Menstrual phases and perceived ADHD medication efficacy:** reports of experiencing unstable effects of medication in relation to menstrual cycle. Some participants reported perceived association between reduced medication efficacy during midluteal phase.	4, unsatisfactory
de Jong et al. (2023) *Case study- qualitative*	*N* = 9, all experiencing premenstrual worsening of ADHD symptoms	*N* = 9 taking psychostimulant medication for ADHD	Range: 22–48	ADHD diagnosis in clinic or confirmed with Diagnostic Interview for ADHD in Adults-5 (DIVA-5)	Interview and self-reported 5-point Likert scale (1: much worsened, to 5: much improved) regarding premenstrual ADHD symptoms following psychostimulant dosage increase	- All participants reported premenstrual worsening of ADHD symptoms, despite medication usage. - **ADHD symptoms:** Following premenstrual increase in dosage of psychostimulant medication for ADHD, all reported improvements (ratings of 4 and 5 on Likert scale). - **Mental health symptoms:** Following premenstrual increase in dosage of psychostimulant medication for ADHD, all participants reported improvement of mood symptoms (ratings of 4 and 5 on Likert scale)	4, unsatisfactory
Roberts et al. (2018), *Observational cohort study*	*N* = 32, regularly cycling females	Not reported	Mean (*SD*): 19.43 (1.38)	High UPPS-P Trait Impulsivity score (high positive urgency, high negative urgency, or high sensation seeking)	ADHD symptom measure: Current ADHD Symptoms Scale: Self-report score Hormone measure: 17-β estradiol, progesterone, testosterone across menstrual cycle	- High negative urgency, high positive urgency and high sensation seeking (ADHD proxy) groups: in higher than average progesterone environments, low estradiol predicted increased ADHD symptoms. - In high sensation seeking group, there were moderated effects of testosterone and estradiol interactions on increasing ADHD symptoms.	6, satisfactory
P. O. Quinn (2005), *Case report- qualitative*	*N* = 1, female	*N* = 1 taking psychostimulant for ADHD	23	ADHD diagnosed by clinician	Qualitative reports of ADHD symptom changes across menstrual cycle	- Reported increase in inattention immediately before menses despite medication use	2, unsatisfactory

Roberts et al. (2018) evaluated interactions between ADHD symptoms and sex hormone concentrations, including estradiol, progesterone, and testosterone, prospectively across the menstrual cycle. Participants were grouped according to scores on the UPPS-P Trait Impulsivity Scale, with participants’ scores +1 standard deviation of the cohort defined as high scorers. Females with high trait impulsivity demonstrated interactive effects of sex hormones and ADHD symptoms: in the context of high progesterone or testosterone environments, low estradiol levels were predictive of an exacerbation of ADHD symptoms. In terms of menstrual cycle phases, the post ovulatory phase (early luteal) was primarily associated with ADHD symptom worsening in females with high trait impulsivity, with the early follicular phase identified as a secondary period of symptom exacerbation.

### Pregnancy

One study explored the relationship between pregnancy and ADHD (Table 4). Baker et al. (2022) explored the relationship between pregnancy and ADHD in a prospective cohort of 25 pregnant females. Females were grouped according to medication use, either: medication continued as normal, change in regime to taking as needed or medication discontinued (Baker et al., 2022). Adult ADHD Investigator Symptom Rating Scale (AISRS) scores and postnatal depression scale scores were collected at three time points throughout pregnancy: <20 weeks pregnant, approximately 24 weeks pregnant, and approximately 36 weeks pregnant. No between-group differences in ADHD symptom scores were determined across pregnancy between medication use groups; however, hyperactivity scores had a larger decrease as pregnancy progressed in medication continuers, compared to discontinuers. Depression scores significantly decreased as pregnancy progressed in medication continuers compared to discontinuers.

**Table 4.** Pregnancy and ADHD.

Author, year, study type	Sample characteristics	Age, mean (*SD*)	ADHD medication use	ADHD diagnosis method	ADHD symptom × hormone measure	Results	Risk of bias
Baker et al. (2022) *Prospective cohort study*	25 pregnant female participants with current or past ADHD psychostimulant use, grouped by maintaining standard medication use through pregnancy (*n* = 12), changed use to as needed (*n* = 8), and discontinued (*n* = 5)	Medication discontinued group:33.2 (4.3), Medication as needed: 32.9 (4.1), Medication maintainers: 33.4 (3.7)	*N* = 25 taking psychostimulant for ADHD	Structured diagnostic clinical interview with psychiatrist, according to DSM-5 criteria	Adult ADHD Investigator Symptom Rating Scale (AISRS) at three time points (<20 weeks pregnant, ~24 weeks pregnant, ~36 weeks pregnant)	- Pregnancy and ADHD medication: No significant between-group differences in AISRS symptom total scores across medication use throughout pregnancy - Pregnancy and hyperactivity: Adjusted mean changes over time showed Hyperactivity AISRS subscores were significantly different between the medication-maintained group compared to the medication-discontinued group as pregnancy progressed (adjusted mean changes: discontinuers = −0.39, maintainers = −2.78, *p* = .0128) - Pregnancy and mental health symptoms: Adjusted mean changes over time showed Edinburgh Postnatal Depression Scale (EPDS) scores were significantly different between medication discontinued group and medication as-needed group (adjusted mean changes: discontinued = 4.32, medication as-needed = −1.01, *p* < .0001), and between medication-discontinued and medication-maintained groups (adjusted mean changes: discontinued = 4.32, medication maintained = −0.65, *p* = .0009)	6, satisfactory

### Endogenous Hormone Levels and Other Hormonal Environments

The remaining three studies explored the associations between ADHD and either endogenous hormone levels or hormonal environments (Table 5).

**Table 5.** Other Hormonal Environments and ADHD.

Author, year, study type	Sample characteristics	Age, mean (*SD*)	ADHD medication use	ADHD diagnosis method	ADHD symptom × hormone measure	Results	Risk of bias
Tsai et al. (2020) *Cross-sectional, cohort study*	26 girls without ADHD, 32 girls with ADHD, between 6 and 12 years of age	Girls with ADHD: 8.7 (1.7) Girls without ADHD: 9.2 (1.9)	Medication free for at least 6 months	Clinical diagnosis by psychiatrist, according to DSM-5 criteria	ADHD diagnosis in relation to serum levels of estradiol and progesterone	-**Sex hormones and ADHD:** No difference between estradiol, progesterone, or testosterone serum levels in ADHD group compared to control	9, very good
Hergüner et al. (2015) *Cross-sectional, cohort study*	40 females with PCOS, 40 females without PCOS	PCOS group: 22.28 (3.68) Control group: 22.33 (3.66)	No current psychotropic medication	N/A	Adult ADHD Self-Report Scale (ASRS) and Wender-Utah Rating Scale (WURS; retrospective symptoms in childhood) scores in people with PCOS compared to without PCOS	- **Adult ADHD symptoms and PCOS:** PCOS group compared to the control group had increased ASRS Total scores (*p* = .012), and Hyperactivity-Impulsivity scores (*p* = .009), however there were no differences in Inattention scores **- Childhood ADHD symptoms and PCOS:** PCOS group compared to control group had increased WURS (childhood ADHD symptoms) Total scores (*p* = .025) and Behavioral Problems/ Impulsivity scores (*p* = .048), however there were no differences in WURS Inattentiveness, School Problems, Depression, or Irritability	7, good
Dinkelbach et al. (2024) *Mendelian randomization study*	4,945 females with ADHD, 16,246 females controls	Not reported	Not reported	Diagnosis by psychiatrist according to ICD-10 criteria	ADHD diagnosis in relation to genetic variants of bioavailable testosterone	- No association between bioavailable testosterone on ADHD risk in females	8, good

#### Childhood Hormone Concentrations

Tsai et al. (2020), measured serum levels of estradiol, progesterone, and testosterone in girls aged 6 to 12 years with and without ADHD diagnoses. No differences in estradiol, progesterone, or testosterone were found between girls with ADHD and without ADHD.

#### Testosterone and ADHD

Dinkelbach et al. (2024), compared genetic variants of bioavailable testosterone in subgroups of females with and without ADHD using a Mendelian randomization study. No causal effects of bioavailable testosterone on ADHD risk were found in females, nor in males.

#### Polycystic Ovary Syndrome (PCOS) and ADHD

Hergüner et al. (2015) investigated ADHD symptoms in a group of 40 females with PCOS, a common endocrine condition associated with hyperandrogenism, compared to a control group of 40 females without PCOS. Inclusion criteria did not include ADHD diagnosis or screening scales. In females with PCOS, Hyperactivity, and Total adult ADHD symptoms scores (ASRS), as well as childhood symptoms of Behavioral Problems/Impulsivity on the Wender-Utah Rating Scale, were significantly increased compared to females without PCOS.

Together, these studies suggest some relationship between endogenous sex hormones and ADHD symptoms in females.

## Discussion

The majority of studies suggest that hormones, or life phases associated with hormonal changes, are associated with a change in ADHD symptoms. However, the small pool of 11 studies discussed in this review demonstrate the limited research specifically investigating ADHD and sex hormones or life phases associated with hormonal changes in females.

### Hormonal Life Phases and ADHD Symptoms

#### Childhood

ADHD is amongst the most prevalent psychiatric conditions in childhood (Sayal et al., 2018). Sex differences in ADHD presentation can be seen from childhood, reflected by the higher prevalence of ADHD in boys compared to girls at an approximate 2.4:1 ratio in the general population (Polanczyk et al., 2007). However, sexual dimorphism in sex hormone levels typically occurs from the ages of eight onwards, meaning differences in ADHD presentation may be reflective of organizational effects of sex hormones (Igarashi et al., 2021). In their study of sex hormones in cohorts of girls from ages 6 to 12 years with and without ADHD, Tsai et al. (2020), found no difference in serum levels of estrogen, progesterone, or testosterone in girls with ADHD compared to girls without ADHD. Thus, whilst sex differences in ADHD presentation amongst girls and boys can be observed, hormonal levels are not specifically different between girls with and without ADHD. Considering the young age of the Tsai et al. cohort, results may also be reflective of the lack of activational effects of sex hormones in prepubertal children. However, given the very limited evidence, more research is needed to understand the relationship between sex hormones and ADHD in childhood.

#### Puberty

Puberty has been recognized as a period of manifestation or exacerbation of ADHD symptoms, as well as comorbid mental health symptoms in females (Eng et al., 2024; Ostojic & Miller, 2016). However, this stance was contradicted by one study in this review: Yuan et al. (2024), reported no association between ADHD and early menarche in Chinese school children. However, results may have been influenced by the method of ADHD diagnosis; ADHD was determined using the Adult ADHD Self Report Scale, which has been validated for use in adults as a screening tool, though was used here as a proxy diagnostic method of ADHD in children. In contrast, two studies included in this review did find evidence for a relationship between puberty and ADHD symptoms (Eng et al., 2023; Ostojic & Miller, 2016). A longitudinal study by Eng et al. (2023), found symptoms of hyperactivity, though not inattention, decreased as females developed pubertally. This aligns with the wider literature, that describes an improvement in externalizing ADHD symptoms with age, and specifically during puberty (Nussbaum, 2012; Vos & Hartman, 2022). Ostojic and Miller (2016) found inattention scores in adulthood were predicted by early pubertal onset. Although the evidence base is not consistent, these studies generally suggest some relationship between puberty, pubertal timing, and ADHD symptoms. Despite perceptions of ADHD improving with age, it has been suggested that puberty is a period of ADHD exacerbation in females, whilst symptoms may improve in males who present with more externalized symptoms (Nussbaum, 2012).

Puberty is a key period of neurodevelopment, coinciding with widespread neuronal proliferation, synapse formation, and elimination (Gurvich et al., 2018). Preclinical rat studies have shown in males, dopamine receptors are overproduced preceding and during puberty, with density reducing into adulthood−this aligns with clinical patterns of ADHD symptomology, as males often experience increased hyperactivity symptoms before and during puberty that alleviate into adulthood (Andersen & Teicher, 2000). Conversely, in female rats, puberty is associated with an increase in dopamine receptors which may relate to an exacerbation of ADHD symptoms (Andersen & Teicher, 2000). This supports recent theories suggesting the organizational effects instigating structural and functional neuronal changes during puberty, in combination with activational effects of fluctuating hormones, may contribute to exacerbate ADHD symptoms in females (Eng et al., 2024). However, these interpretations should be considered preliminary, given no studies reporting associations between ADHD in puberty in this review recruited an all ADHD-diagnosed sample; one study included an over-recruited sample with over 30% of participants diagnosed with ADHD (Eng et al., 2023), and the other did not specify ADHD diagnosis or a proxy as inclusionary criteria (Ostojic & Miller, 2016).

#### Menstrual Cycle

There were four studies included in this review investigating ADHD symptoms across the menstrual cycle. Roberts et al. (2018), reported that the postovulatory/early luteal phase, characterized by low estradiol and high progesterone, was associated with increased impulsivity and hyperactivity symptoms. Two studies (Bürger et al., 2024; de Jong et al., 2023) reported exacerbation of ADHD symptoms including inattention and executive dysfunction, and co-existing mental health symptoms such as depression, irritability and anxiety during the premenstrual/late luteal phase. These studies also explored the reduced efficacy of ADHD medication during the premenstrual/late luteal phase. The late luteal phase is frequently linked with mood disturbances, including symptoms of irritability and fatigue in premenstrual syndrome, to severe sadness and feelings of hopelessness in people with premenstrual dysphoric disorder (Hantsoo & Epperson, 2015). In terms of cognition, the luteal phase has previously been associated with poorer performance in some cognitive domains, however evidence is inconsistent (Le et al., 2020). Investigation of other conditions with similar mechanisms to ADHD, such as schizophrenia, has previously shown specific menstrual cycle phases to be associated with symptom severity fluctuations (Ray et al., 2020). Current theories suggest ADHD symptom-specific hormonal effects across the menstrual cycle; inattention symptoms may be related to decreasing estrogen and moderated by progesterone, whereas hyperactive/impulsivity symptoms may similarly be driven by reducing estrogen levels, though without effect of progesterone (Eng et al., 2024).

#### Pregnancy and Postpartum

The current evidence base investigating ADHD during pregnancy and the postpartum period is very limited, and generally focused on natal development. Baker et al. (2022), explored how medication regime changes affect ADHD and depression symptoms across three time points in pregnancy. Results indicated no differences in ADHD symptom scores between groups who continued ADHD medication as usual, changed medication regime to as-needed, and those who ceased ADHD medication. Findings here may be interpreted as pregnancy and its high estrogen environment positively impacting ADHD symptoms, given adjusted mean differences in ADHD symptoms did not differ between those who ceased medication and those who continued their ADHD medication. However, adjusted mean scores in the hyperactivity subscale were significantly lower for the medication maintainers compared to those who discontinued medication. Given only one study was found to investigate ADHD in pregnancy, and the small group sizes included in the study, more research is required to contextualize these results. The postpartum period has been associated with depression and anxiety, that in addition to psychosocial changes, may be related to changes in sex hormones, including substantial declines in estrogen and progesterone following delivery, as well as changes in cortisol levels that may interplay with cortisol mediated stress responses (Fairbrother et al., 2016; Modzelewski et al., 2023; Szpunar et al., 2021). Our understanding of the relationship between ADHD symptoms and hormones during the postpartum period is limited by the current evidence base; however, comorbid symptoms of anxiety and depression may be more prevalent in postpartum females with ADHD compared to postpartum females without ADHD (Andersson et al., 2023; Dorani et al., 2021).

#### Menopause

Perimenopause is characterized by decreasing and fluctuating estrogen and progesterone levels which stabilize to very low levels in postmenopause. Our literature search found no empirical studies that investigated ADHD during menopause. However, clinicians’ experiences and non-peer reviewed preliminary findings suggest many females report an exacerbation of ADHD symptoms during menopause (Antoniou et al., 2021; Wasserstein et al., 2023). There is evidence of females with ADHD experiencing greater climacteric symptoms during menopause, which may be indicative of hormonal vulnerabilities related to mechanisms of ADHD in females (Dorani et al., 2021). In similar conditions to ADHD such as autism spectrum conditions, menopausal symptoms have been associated with increased autistic traits (Groenman et al., 2022). Menopause is associated with cognitive symptoms in many females, with brain fog and executive dysfunction frequently reported, and potentially related to the fluctuating and declining levels of estrogen (Conde et al., 2021; Khadilkar & Patil, 2019). It may therefore be challenging to disentangle exacerbation of ADHD symptoms in menopause from the brain fog symptoms more directly associated with menopause itself (Pines, 2016). One study found lisdexamfetamine, a psychostimulant used in treatment of ADHD, improved Brown Attention Deficit Disorder Scale scores in a community sample of menopausal females experiencing executive dysfunction (although ADHD was not specifically measured; Epperson et al., 2015). A similar study in menopausal females found executive functioning to improve with lisdexamfetamine administration (Shanmugan et al., 2017). Psychostimulants have demonstrated beneficial effects in non-clinical samples, and therefore improvement in cognitive functioning with their use may not be indicative of the presence of ADHD (Bagot & Kaminer, 2014). However, improvement of ADHD-like symptoms in menopausal people with psychostimulant use reflects similarities between symptomologies of ADHD and menopausal cognitive dysfunction.

### Estrogens, Progesterone, and ADHD in Females

The hormonal milieu experienced by females throughout life may impact ADHD symptoms. Specifically, low estrogen environments appear to be most often associated with ADHD symptom exacerbation, although further investigation of these relationships is required. ADHD etiology is complex and not fully understood, however dysfunction of dopaminergic pathways have consistently been reported in people with ADHD (Faraone et al., 2024). Estrogen has been demonstrated to increase dopamine and serotonin synthesis and receptor levels, as well has limiting reuptake through inhibition of monoamine oxidase (Del Río et al., 2018). Regions of interest in ADHD pathophysiology including the basal ganglia and prefrontal regions may be particularly sensitive to the effects of estrogen. For example, dopamine neurons in the basal ganglia have shown expression of estrogen receptors, and the relationship between estradiol and dopamine have demonstrated effects in prefrontal cortex functions (Bendis et al., 2024; Jacobs & D’Esposito, 2011). Lower and fluctuating estrogen levels may therefore impact regulation of dopamine synthesis and activity. Given the existing dysregulation of dopaminergic pathways in ADHD, further fluctuations may exacerbate mechanisms of ADHD pathophysiology, and/or alter the efficacy of stimulant medication, leading to an increased severity of ADHD symptoms during times of hormonal change, such as the luteal phase of the menstrual cycle.

### Androgens and ADHD in Females

Estrogen and progesterone are the key neurosteroids in females; however, androgens such as testosterone also exert effects. Two studies included in this review explored ADHD in females in the context of androgens. Dinkelbach et al. (2024), investigated this relationship directly through analysis of genetic variants of bioavailable testosterone in females with ADHD compared to females without ADHD, finding no significant differences. The relationship between androgens and ADHD in females was also investigated by Hergüner et al. (2015), though this was achieved indirectly through comparison of ADHD scores in a group of females with and without PCOS. Females with PCOS, who have characteristically higher levels of androgens, were demonstrated to have higher hyperactivity/impulsivity scores in ADHD scales compared to controls (Hergüner et al., 2015).

In males, testosterone has been investigated in the context of ADHD, showing mixed effects (Martel et al., 2009; Rogne & Hassel, 2022). Prenatal androgen exposure in ADHD has more commonly been studied, with indicators of higher testosterone in utero associated with greater ADHD symptoms. There is limited research of the relationship between cognition in females and testosterone; one study found testosterone administration had no cognitive effects in females with low testosterone after surgical menopause (Huang et al., 2015). Conflicting evidence here may be indicative of symptom specific effects of androgens in females. Specifically, higher androgen levels were not associated with inattention symptoms, nor was bioavailable testosterone associated with ADHD risk. However hyperactivity/impulsivity symptoms were higher in females with higher androgens (PCOS) compared to control females. Similarly, males who have higher circulating androgens compared to females, also have higher hyperactivity/impulsivity symptoms (Young et al., 2020). These results may therefore suggest androgens are associated with hyperactive symptoms of ADHD specifically, though more research is required to explore this relationship.

### Limitations

There are several limitations that should be considered when interpreting these findings. Primarily, only 11 studies met criteria for inclusion, with varying ADHD diagnostic criteria (only 6 studies included participants with a diagnosis of ADHD), diverse ADHD outcome measures and different hormonal environments were included in this review. Study types included qualitative interviews, prospective designs, and cross-sectional cohort studies, and often included small sample sizes. The variability of methodological aspects in combination with the often small sample sizes may limit accuracy of our conclusions based on the synthesis of these studies, and reduce generalizability to the population of females with ADHD. Findings are also limited by the mixed quality of studies; although most were satisfactory, the novelty of the research area means 4 of the 11 studies included were classified as having an unsatisfactory risk of bias. Three of these studies (Bürger et al., 2024; de Jong et al., 2023; P. O. Quinn, 2005) received an unsatisfactory risk of bias classification due to their qualitative nature, lack of statistical analysis, inclusion of small sample sizes, and no experimental control. Therefore, conclusions drawn from these studies may provide direction for future studies, though should be considered with acknowledgment of potential biases. The fourth study classified as having an unsatisfactory risk of bias was due to lack of clarity in the ascertainment of ADHD diagnosis and details of sampling strategy and selection of sample size (Yuan et al., 2024). Whilst these factors limit the conclusions that can be drawn from this systematic review, our findings suggest a potential relationship between ADHD and sex hormones in females.

### Future Research Directions

This review suggests that females with ADHD experience changes in symptoms that may reflect changes in endogenous sex hormones, such as during puberty, pregnancy, across the menstrual cycle, and during menopause. Future research is warranted to investigate the nature of the association between sex hormones and ADHD symptoms, as well as co-existing mental health symptoms at different life stages that can include self-report methodology and prospective studies to enhance understanding of how ADHD symptoms, characteristics, and symptoms may correspond to life stages associated with hormonal changes. In addition, to advance our understanding of ADHD in females, research that seeks to understand the mechanisms underlying how sex hormones may influence ADHD symptoms is essential. This should include a multi-disciplinary approach that combines assessments of hormone levels (and/or assessments at times of hormonal change) with neurocognitive, brain imaging, genetic, or neurophysiological investigations. To progress this field, future research that involves females with ADHD should provide detailed descriptions of the assessment and diagnostic processes that were used to establish ADHD diagnosis. This should include information on whether an interview was conducted or a screening tool was completed; who conducted the interview; whether there was an informant or other corroborative information to help confirm ADHD (eg. school reports or evidence to suggest symptoms were present prior to the age of 12 years), in line with DSM-5 as well as recommendations outlined in clinical practice guidelines. Future research should consider the role of ADHD medications, and hormonal contraceptives/hormonal therapies. This should include reporting medications and dose in combination with information about the use of any hormonal contraceptives or hormonal therapies. Collectively, such a rigorous and comprehensive approach will aid in the understanding of the underlying mechanisms of ADHD in females, facilitating development of a holistic understanding of ADHD in the unique hormonal contexts in females.

### Clinical Implications

Recognizing potential influences of sex hormones on ADHD symptoms in females may have key implications to clinical management and treatment of ADHD. Increased awareness of hormonal phases considered to be linked to changes in ADHD symptomology in females may better prepare health professionals to identify ADHD symptoms and improve diagnosis. Additionally, this area of research may provide direction for future therapies for females who experience exacerbation of ADHD symptoms during periods of hormonal fluctuations, particularly for those who experience decreased efficacy of their ADHD medication at different points in the menstrual cycle or during the menopause transition years. Therefore, clinicians should be encouraged to consider a patient’s hormonal life phase and ask patients whether they are aware of any associations between their ADHD symptoms or any co-existing mood symptoms that may fluctuate in association with changes in hormone levels (e.g., across the menstrual cycle, in perimenopause, in pregnancy, or following the introduction or cessation of hormonal therapies). This may help guide treatment options, which could include premenstrual adjustment of psychostimulant dose (as per de Jong et al., 2023) or the potential use of hormonal therapies to stabilize endogenous hormone levels, currently explored in premenstrual dysphoric disorder (Appleton, 2018), or enhance fluctuating or depleting hormones, currently explored in menopausal depression (Herson & Kulkarni, 2022). Hence recognizing any links between hormonal changes and ADHD experiences may help improve management of ADHD during periods such as menopause and the late luteal phase.

## Conclusion

This systematic review generally supports a relationship between sex hormones and ADHD symptoms in females, however, the current evidence base is extremely limited. We emphasize the need for further investigation into the relationship between sex hormones and ADHD symptoms in females. Despite this, the studies reviewed indicate that there is an ADHD experience that is unique to females, and highlight the need for female-specific research in ADHD. Knowledge of ADHD over the life course of females is integral to improving its management and treatment. Recognizing potential influences of sex hormones on ADHD symptoms in females may direct future therapies, specifically targeting the hormonal elements of ADHD exacerbations during phases such as menopause and the luteal phase of the menstrual cycle.

## Supplemental Material

sj-docx-1-jad-10.1177_10870547251332319 – Supplemental material for ADHD and Sex Hormones in Females: A Systematic Review Supplemental material, sj-docx-1-jad-10.1177_10870547251332319 for ADHD and Sex Hormones in Females: A Systematic Review by Elyssa Osianlis, Elizabeth H. X. Thomas, Lisanne Michelle Jenkins and Caroline Gurvich in Journal of Attention Disorders

## Notes

1. Sex refers to physiological and biological characteristics of males, females and intersex people, whereas gender is based on social constructs, including “men” and “women”; sex and gender identity are not necessarily the same within each person.

2. Given the focus on endogenous hormones in females here, the term “females” is used to describe people assigned female at birth. *was used to truncate words to search for multiple word endings.
