# Supplementary material for: ADHD and Sex Hormones in Females: A Systematic Review
Source: J Atten Disord. 2025 Apr 18;29(9):706–23. doi: 10.1177/10870547251332319 (PMC12145478; doi:10.1177/10870547251332319)
Supplement: sj-docx-1-jad-10.1177_10870547251332319 – Supplemental material for ADHD and Sex Hormones in Females: A Systematic Review [file sj-docx-1-jad-10.1177_10870547251332319.docx]

# ***Supplementary Material 1 Risk of Bias Criteria***

| **Selection** (Maximum 4 stars)   1. Representativeness of the Exposed Cohort    1. Truly representative of average in target population * (all subjects or random sampling)    2. Somewhat representative of average in target population * (non-random sampling)    3. Selected group of users    4. No description of sampling strategy 2. Sample size    1. At least 30 participants in ADHD group *    2. Not justified 3. Ascertainment of ADHD diagnosis    1. ADHD diagnosis *    2. structure interview/ scale scores *    3. self report    4. no description 4. Ascertainment of hormonal characteristics    1. Details of hormonal status provided (e.g. validation of phase of menstrual cycle, pubertal phase)*    2. No details/ validation of hormonal characteristics provided |
| --- |
| **Comparability** (Maximum 2 stars)   1. Comparability of cohorts on the basis of the design or analysis    1. study controls for most important factor (ADHD medication usage, hormonal disorders/ treatment use)*    2. the study controls for any additional factor *    3. inadequate degree of control |
| **Outcome** (Maximum 3 stars)   1. Assessment of outcome    1. validated method to measure ADHD symptoms **    2. non validated measurement tool, but method is available or described *    3. no description of measurement tool 2. Statistical test    1. test used to analyses data clearly described and appropriate, and measurement of association is presented, including probability level (p value)*    2. No statistical test, or the test in not appropriate, not described, or incomplete |
| **Total score:** (Possible scores 0-9) |
